# Relapsing Eosinophilia in a Severe Allergic Asthma Patient on Biological Therapy

**DOI:** 10.3390/jcm13123402

**Published:** 2024-06-11

**Authors:** Oana Raduna, Bianca Oprescu, Stefan Mihaicuta, Stefan Frent

**Affiliations:** 1 “Victor Babes” Infectious Diseases and Pulmonology Clinical Hospital Timisoara, 300310 Timisoara, Romania; oanabigiu@gmail.com (O.R.); stefan.mihaicuta@umft.ro (S.M.); frentz.stefan@umft.ro (S.F.); 2 Centre for Research and Innovation in Precision Medicine of Respiratory Diseases, Department of Pulmonology, “Victor Babes” University of Medicine and Pharmacy Timisoara, 300041 Timisoara, Romania

**Keywords:** hyperosinophilia, benralizumab, omalizumab, severe asthma, biological therapy

## Abstract

**Background:** Severe asthma often remains uncontrolled despite optimized inhaled treatment. The rise of biologic therapy in severe asthma represented a major advance for the disease management. However, correct phenotyping and monitoring of severe asthma patients is key to the success of targeted biologic therapy. **Materials and Methods:** We present the case of a 63-year-old female, never a smoker, diagnosed with asthma at the age of 45 and associated persistent mild rhinitis, without other notable comorbidities. She was prescribed medium-dose ICS/LABA, administered inconstantly in the first years after the diagnosis, with poor overall control of the disease. After several exacerbation episodes, treatment compliance improved, but the control of the disease remained poor despite adding an antileukotriene. In January 2019, she presented an exacerbation episode requiring treatment with oral corticosteroids (OCS) and she was afterwards put on high-dose ICS/LABA and continued the antileukotriene. She was referred for a skin allergy test, which revealed mild sensitization to Dermatophagoides pteronyssinus and farinae, with a total IgE level of 48.3 IU/mL. The blood eosinophil level was 270 cells/mm^3^. The lung function was variable, going from mild impairment to severe fixed obstruction during exacerbations. Despite optimized inhaled treatment, good adherence and inhaler technique, and allergen avoidance strategies, asthma control was not achieved, and she continued to experience severe episodes of exacerbation requiring OCS. **Results:** In October 2019, she was initiated on biologic therapy with omalizumab, which allowed asthma control to be achieved and maintained for 18 months, with preserved lung function, good symptom control, no exacerbations and slightly elevated blood eosinophil level (340–360 cells/mm^3^). In April 2021, she started experiencing exacerbation episodes requiring OCS (three episodes within 6 months), with a progressive increase in blood eosinophil level (up to 710 cells/mm^3^), and progressive deterioration of asthma control and lung function, despite continuation of previous therapy. A specific IgE test against Aspergillus was negative, and total IgE level was 122.4 IU/mL. In December 2021, the patient was switched from omalizumab to benralizumab. Asthma control was again achieved, lung function improved significantly and the patient did not experience any other exacerbation episodes up until today, which allowed for a reduction in ICS dose. Intriguingly, a relapsing eosinophilia was also noted under anti-IL5-R treatment prior to the dose administration, but with preserved asthma control. **Conclusions:** This case underscores the pivotal role of meticulous phenotyping in severe asthma management on one side, and careful monitoring of patient evolution and possible side effects of treatment on the other side. By showcasing how diverse inflammatory pathways can coexist within a single patient and impact treatment outcomes, it highlights the necessity of tailored biologic therapy for sustained control.

## 1. Introduction

Asthma, recognized as a major noncommunicable disease (NCD) by the World Health Organization (WHO), impacts all age groups, notably affecting children worldwide. It involves inflammation and constriction of the lungs’ small airways, leading to symptoms like coughing, wheezing, and chest tightness. In 2019, around 262 million people suffered from asthma, contributing to 455,000 deaths globally. It typically begins in childhood, with symptoms appearing before age 5, showing higher prevalence among boys, later shifting to impact women more in adulthood. Urban and developed areas exhibit higher asthma rates, with socio-economic factors, genetics, and environmental exposures playing significant roles. Lower socioeconomic status is linked to increased asthma rates due to greater exposure to indoor allergens and pollutants. Certain occupations carry a higher risk of occupational asthma. These demographic patterns underscore the complex interplay of genetic, environmental, and social factors influencing asthma prevalence. Asthma imposes a considerable societal burden, incurring substantial healthcare costs, productivity losses, and diminishing individuals’ quality of life. Direct expenses include medical services, medications, and hospitalizations, while indirect costs stem from reduced productivity at work or school. Notably, 40% of asthma patients have moderate to severe disease [1].

According to the definition by the Global Initiative for Asthma (GINA), severe asthma patients may exhibit persistent symptoms despite adhering to high-dose inhaled corticosteroids and additional controllers, and may present with reduced lung function that remains impaired despite appropriate treatment. This condition is characterized by frequent exacerbations, nocturnal symptoms, and poor quality of life. It can further be categorized into severe eosinophilic asthma, marked by elevated blood eosinophil levels, and severe non-eosinophilic asthma, which is associated with neutrophilic or paucigranulocytic inflammation. Such patients require specialized assessment and tailored management, often incorporating biologic therapies, in order to achieve better control and enhance their quality of life [2].

Asthma phenotypes and endotypes are two critical concepts that contribute to a more nuanced understanding of the heterogeneity within the asthma population. Phenotyping involves categorizing individuals based on observable clinical and biological characteristics. Clinical phenotypes, such as allergic, non-allergic, early-onset, or late-onset asthma, provide a practical framework for classifying patients. On the other hand, endotyping delves deeper into the molecular and biological mechanisms involved in the asthma pathogenesis, by identifying specific pathways and biological markers associated with the disease. For example, eosinophilic and neutrophilic endotypes focus on the predominant inflammatory cells in the airways, offering insights into potential targeted therapies [3].

Induced sputum analysis plays a crucial role in classifying asthma phenotypes based on specific inflammatory patterns identified in the respiratory secretions. This classification includes eosinophilic, neutrophilic, mixed granulocytic, and paucigranulocytic phenotypes, each reflecting distinct inflammatory responses. The presence of elevated eosinophils suggests a Th2-mediated response, often associated with allergic triggers, while elevated neutrophils indicate a non-Th2 or neutrophilic response, potentially linked to non-allergic triggers or infections. The mixed granulocytic phenotype highlights the heterogeneity within asthma, showcasing a combination of inflammatory mechanisms. Additionally, the assessment of cytokine profiles offers insights into molecular signaling associated with different asthma phenotypes [3].

The synergy between phenotyping and endotyping is transforming asthma management towards a more personalized approach.

The introduction of biologic therapy in clinical practice completely changed the horizon of severe asthma management. The concept of asthma remission slowly begun to take shape, as reports of its occurrence gradually emerged.

Omalizumab, a recombinant humanized monoclonal antibody, is strategically designed to target and neutralize immunoglobulin E (IgE). Primarily employed in the management of severe allergic asthma and chronic idiopathic urticaria, this therapeutic agent interrupts the IgE pathway by binding specifically to the constant region (FcεRI) of IgE antibodies. Administered subcutaneously, omalizumab inhibits the IgE-mediated release of inflammatory mediators from mast cells and basophils, contributing to a reduction in airway inflammation and improving asthma symptoms. Its notable efficacy is particularly evident in cases of severe allergic asthma that are unresponsive to conventional treatments. Beyond asthma, omalizumab has proven effective in treating chronic idiopathic urticaria. This targeted approach highlights omalizumab’s role as a valuable tool in modulating the immune response and improving clinical outcomes in challenging-to-treat allergic conditions, underscoring its importance in personalized medical interventions.

Benralizumab is a monoclonal antibody designed to target the interleukin-5 receptor (IL-5R), a critical component in the eosinophilic inflammation cascade. This type of inflammation is characterized by elevated levels of eosinophils in both the bloodstream and the airways. By binding to the IL-5R found on the surface of eosinophils, benralizumab initiates a process called antibody-dependent cell-mediated cytotoxicity (ADCC), leading to the destruction of eosinophils. As a result of this eosinophil depletion, there is a notable reduction in airway inflammation and its associated negative effects. This unique mechanism of action provides a targeted strategy for mitigating inflammation driven by eosinophils, which in turn enhances asthma control and decreases the occurrence of exacerbations in individuals with severe eosinophilic asthma [4].

We present the case report of a non-smoker, middle-aged female patient with severe allergic asthma, in whom relapsing blood eosinophilia developed both after initial remission on biological therapy with anti-IgE monoclonal antibody and after the switch to an anti-IL5-R agent. Our aim was to emphasize the importance of carefully phenotyping severe asthma in order to administer the best available targeted therapy, by illustrating how different inflammatory pathways can co-exist in the same patient and may influence the clinical evolution under treatment. Additionally, our goal was to emphasize the importance of carefully monitoring the occurrence of possible adverse reactions in patients under biologic treatment.

## 2. Case Presentation

A 63-year-old female non-smoker patient presented with a history of biomass exposure, associated persistent mild rhinitis and passive tobacco smoke exposure, without notable coexisting medical conditions. The patient was diagnosed with asthma in the year 2004 and is currently under maintenance treatment with Budesonide/Formoterol 320/9 mcg 1 puff b.i.d., demonstrating appropriate inhalation technique. Because of poor asthma control, the patient is also prescribed Montelukast as an additional controller medication.

On 26 November 2018, the patient experienced a severe exacerbation episode that prompted a series of investigations. These encompassed a comprehensive assessment including clinical, radiological, biological, and functional assessments. The onset of asthma symptom worsening was two weeks earlier and showed no signs of improvement despite nonspecific symptomatic interventions. The presenting symptoms were non-productive cough, particularly worse during nocturnal hours, inspiratory dyspnea and wheezing. Notably, there was an absence of fever, the patient’s appetite was unaffected, and the chest X-ray was clear. Laboratory findings revealed an eosinophil count of 270/mmc, an erythrocyte sedimentation rate (ESR) exceeding twice the normal value, slightly elevated C-reactive protein (CRP) level, while urea, creatinine, and glucose levels remained within normal ranges. Spirometry unveiled evidence of a moderate distal obstructive syndrome (decreased FEF25-75), with a normal forced expiratory volume in 1 s (FEV1), forced vital capacity (FVC) and FEV1/FVC ratio. To manage this flare-up, oral Prednisone was prescribed for a duration of 7 days and consultation with an allergologist was advised for further assessment and management.

On 14 January 2019, a subsequent severe exacerbation episode occurred in the context of poorly controlled asthma symptoms over the preceding 2-month period, despite the appropriate usage of maintenance medications, including regular use of Budesonide/Formoterol 320/9 mcg 1 puff b.i.d., Montelukast 10 mg o.d. and Ventolin 100 mcg p.r.n. Clinical examination revealed bronchial rales, and spirometry testing exhibited severe fixed obstructive ventilatory dysfunction. The forced expiratory volume in 1 s (FEV1) was measured at 35% of the predicted value, with a FEV1/FVC ratio < 0.7. The bronchodilator test demonstrated reversibility, with a 250 mL and 33% increase in FEV1 15–20 min after the administration of 400 mcg salbutamol. After this exacerbation episode, a decision was made to step up the controller medication to a high dose of inhaled corticosteroid (ICS): Fluticasone/Salmeterol 50/500 mcg 1 puff b.i.d., alongside a 10-day course of oral Prednisone. Furthermore, it was advised that the patient seek consultation with an allergologist for further evaluation and comprehensive allergy tests.

On 21 January 2019, the patient was seen by an allergologist, and she underwent a skin prick test for common aeroallergens. The results of the test unveiled sensitization to dust mites, specifically Dermatophagoides pteronyssinus and farinae species. The primary recommendation involved implementing measures to decrease the presence of dust mites within the patient’s living environment. Additionally, the patient was recommended an assessment of both total and specific immunoglobulin E (IgE) levels, targeted at assessing the patient’s immune response to dust mites, particularly the allergenic proteins Der P1-D1 and Der F1-D2.

On 4 September 2019, the patient experienced a recurrence of asthma exacerbation symptoms, triggered by an ongoing upper respiratory tract infection. The patient’s complaints included an exacerbated cough lasting for a week, accompanied by mucopurulent expectoration, heightened dyspnea, and increased wheezing. Nocturnal awakenings have been noted and, possibly, low-grade fever. Concurrently, the patient experienced watery rhinorrhea and frequent sneezing. The Asthma Control Test (ACT) score was 16, indicating suboptimal asthma control. Spirometry testing revealed evidence of moderate obstructive ventilation impairment, with a forced expiratory volume in 1 s (FEV1) measuring 64% of the predicted value and FEV1/FVC ratio > 0.7. The patient had been prescribed antibiotic and symptomatic treatment by the family doctor.

A re-assessment of the patient’s condition was conducted on 10 September 2019. Over the previous three days, the patient had reported a progression in symptoms, including increased dyspnea, heightened wheezing, productive cough, and early morning awakenings prompted by asthma symptoms. Upon clinical examination, bilateral disseminated bronchial rales were detected. A new prescription of oral Prednisone could not be avoided in face of the evidence of another asthma flare-up.

On 30 October 2019, the patient presented with ongoing symptoms, including a persistent cough that was intermittently productive with mucous expectoration, recurrent dyspnea, and occasional wheezing. There had been no additional respiratory infections since the patient’s previous visit. The Asthma Control Test (ACT) score remained at 16, indicating suboptimal asthma control. As part of the functional evaluation, a mild obstructive ventilatory dysfunction was noted, with a forced expiratory volume in 1 s (FEV1) reaching 77% of the predicted value and a normal FEV1/FVC ratio. The total immunoglobulin E (IgE) level was 48.3 IU/mL.

Based on the information provided, a diagnosis of severe asthma was established, which remained uncontrolled despite maximal inhaled therapy corresponding to the Global Initiative for Asthma (GINA) therapeutic step 5 and despite good adherence and inhalation technique and appropriate management of the comorbidities. The patient’s medical history demonstrated a recurrence of three exacerbation episodes requiring administration of oral corticosteroids within the past year.

Further management of this condition involved the initiation of the treatment with Omalizumab, the only biologic agent available in our country at that time, administered subcutaneously at a dosage of 150 mg every month. The inhalation therapy was maximized to receive Beclomethasone/Formoterol 200/6 mcg DPI, with a dosing regimen of 2 puffs b.i.d.

According to the national protocol for the prescription of Omalizumab, an assessment of the patient’s condition was conducted after a period of 4 months, on 25 February 2020. The patient’s symptoms included rare coughing with mucous expectoration and no reporting of dyspnea, wheezing, or nocturnal awakenings. Additionally, there had been no instances of moderate or severe exacerbation episodes since the initiation of biologic therapy, and the patient’s Asthma Control Test (ACT) score had improved to 23. Spirometry testing showed mild obstructive ventilatory dysfunction, including an FEV1 at 70% of the predicted value and a normal FEV1/FVC ratio.

Considering the favorable clinical evolution, the lack of exacerbations, and a preserved lung function, a decision was made to continue the treatment with Omalizumab, in addition to maintenance therapy with inhaled Beclomethasone/Formoterol 200/6 mcg DPI, 1 puff b.i.d. and oral Montelukast 10 mg o.d., using Ventolin 100 mcg p.r.n. as rescue medication. Subsequently, over a span of 12 months, the patient showed a favorable clinical evolution under anti-IgE biologic therapy, with no exacerbation episodes, good symptom control and preserved lung function (Table 1).

However, this favorable trend was reversed after 16 months, with a loss of asthma control, a significant decrease in lung function, and a resurgence of exacerbations coinciding with an important increase in blood eosinophil levels (Table 2).

The patient began to experience exacerbation episodes requiring oral corticosteroids (three episodes within six months), accompanied by a progressive increase in blood eosinophil levels (710 cells/mm^3^), and a deterioration in asthma control and lung function, despite the maintenance of previous therapy. The chest X-ray did not demonstrate the presence of pulmonary infiltrates.

On 21 December 2021, a diagnosis of severe eosinophilic asthma was established, uncontrolled despite Global Initiative for Asthma (GINA) step 5 maintenance treatment, including a biologic agent. Consequently, the switch to a different biologic therapy was taken into consideration. In this context, in order to test for the presence of allergic bronchopulmonary aspergillosis (ABPA), we performed a specific IgE test for Aspergillus, which was negative, and the total IgE level was measured at 122.4 IU/mL.

The new treatment strategy involved cessation of previous biologic therapy (Omalizumab) and the initiation of Benralizumab as an add-on therapy, in conjunction with the continued use of Beclomethasone/Formoterol DPI 200/6 mcg, 2 puffs b.i.d. The patient was also prescribed oral Montelukast at a daily dosage of 10 mg and inhaled Salbutamol 100 mcg p.r.n. as rescue medication. The therapeutic response was once again favorable, with remission of asthma symptoms, significant improvement in lung function and cessation of exacerbation episodes. This favorable trend was maintained until the present, allowing the lowering of ICS doses. However, a relapsing blood eosinophilia was again noted, just prior to the administration of anti-IL5-R therapy, but with preserved asthma control (Table 3).

## 3. Discussion

The reported case study is of interest for several reasons. Beyond the exemplification of the interplay between different mechanisms in asthma pathogenesis, a relapsing eosinophilia under two different biologic agents may reflect the underlying T2 inflammatory pathways, as well as raise the question of a possible side effect of the treatment or the presence of a concomitant disease.

Asthma exhibits a remarkable level of diversity, encompassing various clinical, functional, and molecular phenotypes underpinned by complex immunopathological mechanisms. Additionally, phenotypic transitions and overlaps are observed in a substantial 50–70% of individuals with asthma, highlighting the intricate and multifaceted nature of the disease [3].

The phenomenon of phenotype overlap underscores the heterogeneity within the severe asthma population. Individuals with severe asthma often display a mix of Th2-mediated and non-Th2-mediated inflammation, involving both eosinophilic and neutrophilic responses. This diversity poses challenges in treatment decisions, as therapies targeting specific pathways may not comprehensively address the multifaceted inflammatory landscape. Moreover, the overlap reflects the dynamic nature of asthma, where environmental factors and individual variations contribute to the evolution of the disease phenotype over time [3].

Understanding phenotype overlap in severe asthma is crucial for advancing personalized and targeted therapeutic strategies. It requires a shift from a one-size-fits-all approach to a more nuanced and individualized treatment paradigm. Biomarkers and advanced diagnostic tools play a pivotal role in identifying the specific inflammatory patterns contributing to phenotype overlap, guiding clinicians in tailoring interventions that address the unique characteristics of each patient’s asthma. Ultimately, unraveling the complexities of phenotype overlap in severe asthma holds the key to optimizing patient care, improving treatment outcomes, and advancing our overall comprehension of this challenging respiratory condition.

The case discussion sheds light on the complex journey of managing severe eosinophilic asthma. Initially, a promising improvement in asthma control was achieved with omalizumab, an anti-IgE biologic therapy, but later, the challenges of fluctuating asthma control and recurring exacerbations underscored the dynamic nature of this condition. This emphasizes the importance of tailoring treatments to the individual, closely monitoring progress, and being adaptable in managing severe eosinophilic asthma. The switch to benralizumab, a different biologic targeting the interleukin-5 receptor, not only shows potential for positive responses but also reflects ongoing efforts to improve outcomes in severe asthma management.

Furthermore, the case highlights the convergence of at least two different mechanisms contributing to type 2 inflammation in severe asthma. While omalizumab initially helped control allergic sensitization mechanisms, leading to a stable period of 1.5 years, subsequent challenges in asthma control suggested the presence of another pathway sustaining type 2 inflammation. This alternative mechanism, likely involving alarmins and innate lymphoid cells type 2 (ILC2), is demonstrated by increasing levels of eosinophils in the blood. The success of benralizumab, going beyond traditional markers of allergic sensitization, suggests a crucial role for eosinophils and ILC2 cells in maintaining type 2 inflammation in this patient. This underscores the need for careful and personalized treatment strategies suited to the varied immunological landscape seen in severe asthma. Continued research and clinical experience are crucial to figuring out the best sequence of biologic therapies and refining strategies to provide lasting benefits for those dealing with severe eosinophilic asthma.

Interestingly, under anti-IL5 receptor therapy, the patient’s evolution was relatively stable, with no episodes of exacerbation requiring systemic corticosteroids; however, we noticed a repeated and significant increase in blood eosinophil level (up to 300 cells/microL) prior to the administration of benralizumab, which is quite unusual for patients under this type of therapy, but without deterioration of asthma control. This phenomenon raises several points for discussion. First, we could question the efficacy of benralizumab in destroying the eosinophils in this patient, possibly by the production of antibodies against this biologic therapy. Second, given the previous experience with an anti-IgE biologic agent which allowed a good control of asthma for 1.5 years, we could also presume that the allergic mechanisms may be responsible for the increase in blood eosinophil level, despite continued therapy with an anti-IL5 receptor agent. Furthermore, this could raise the question of whether the association of two different biologic drugs targeting different pathophysiological mechanisms could be envisaged in patients with evidence for these mechanisms. Third, another important question is the temporal relationship between the pathophysiological mechanisms underpinning the airway inflammation in this patient. Most likely, the allergic pathway developed first, and the pathway involving alarmins and ILC2 emerged later, under the effect of repeated viral infections. During the lifetime of asthmatic patients who develop a T2 immune and inflammatory response, diverse environmental stimuli can induce modifications of the pre-existing inflammatory profile, ending up in mixed molecular pathways and phenotype overlap [3].

The clinical path of benralizumab unfolds in the context of robust trials that provide substantial evidence of its efficacy. Clinical trials like SIROCCO and CALIMA have unequivocally demonstrated benralizumab’s capability to significantly lower exacerbation rates and enhance lung function among patients grappling with severe eosinophilic asthma [5,6]. Worth mentioning are the more recent GALATHEA and TERRANOVA trials, which have further extended the comprehension of benralizumab’s therapeutic potential across a broader spectrum of patients [7,8]. However, they failed to demonstrate a clear benefit in COPD patients with eosinophilic inflammation. Nevertheless, these trials collectively underscore the efficacy of benralizumab in addressing the underlying inflammation and its subsequent impact on asthma severity, thus paving the way for more tailored and effective management strategies in severe eosinophilic asthma cases.

The overlap of inflammatory phenotypes in eosinophilic asthma is a complex and dynamic aspect of asthma pathogenesis. Previous research highlights a significant overlap among atopic, eosinophilic, and T2-high asthma phenotypes within a general asthma population, with a substantial proportion of children and adults exhibiting characteristics of eosinophilic asthma (ranging from 21% to 78%, depending on eosinophil level cutoff), atopic disease (63% of children, 61% of adults), and T2-high asthma (48% of children, 37% of adults). Furthermore, higher eosinophil cutoff points correlate with increased classification of eosinophilic asthma as atopic or T2-high, while fewer cases of atopic or T2-high asthma meet criteria for eosinophilic asthma [9]. Additionally, instability of phenotypes defined by biomarkers, particularly eosinophils, was reported in adult asthmatics over a one-year period, with significant changes observed in phenotype clustering based on sputum cellularity [10]. The heterogeneity of severe asthma is also underscored by the identification of multiple phenotypes based on the presence or absence of the biomarkers currently used to characterized the T2-high inflammation, such as blood eosinophil level, IgE, and FeNO [11]. Cluster analysis further illustrates substantial biomarker overlap in severe asthma, identifying distinct clusters with unique clinical features linked to different inflammatory pathway activations [12]. Exploration of T2 inflammatory marker co-expression in severe asthma reveals high heterogeneity in expression patterns associated with allergic sensitization and comorbidities [13].

Alongside conventional strategies for treating severe asthma exacerbations (SAEs), such as inhaled β-agonists, anticholinergics, and systemic corticosteroids, ketamine represents another pharmacological option. However, evidence regarding the effectiveness of ketamine in refractory SAEs remains inconclusive. Well-designed multicenter randomized controlled trials are needed to clarify its role in this context [14].

Omalizumab and benralizumab, distinct in their mechanisms, exhibit differential effects on blood eosinophilia in the treatment of asthma. Omalizumab, targeting immunoglobulin E (IgE), is efficacious in allergic asthma, often resulting in reductions in blood eosinophil levels by modulating allergic inflammation, particularly in patients unresponsive to standard therapy [15,16]. In contrast, benralizumab, specifically tailored for severe eosinophilic asthma, induces rapid eosinophil depletion via interleukin-5 receptor alpha (IL-5Rα) targeting, leading to profound and nearly complete reductions in blood eosinophil counts [5,6]. Clinical trials consistently demonstrate the efficacy of benralizumab even in patients refractory to conventional therapies, highlighting its direct and potent impact on eosinophilia in severe asthma. These observations underscore the tailored therapeutic approaches of omalizumab and benralizumab in addressing distinct asthma phenotypes based on their effects on blood eosinophilia.

Despite the varied findings documented in the literature, we present a compelling case where a patient, after receiving treatment with omalizumab followed by benralizumab, exhibited persistently elevated levels of blood eosinophils, albeit without experiencing exacerbation episodes. This case underscores the complexity and heterogeneity of asthma presentations and treatment responses. Despite the established efficacy of these medications in modulating eosinophilic inflammation when administered separately, exceptions such as this case highlight the importance of vigilant monitoring and individualized patient management. While the majority may respond favorably to standard treatments, exceptions will inevitably arise, emphasizing the necessity for ongoing assessment and flexibility in clinical decision making to optimize patient outcomes.

Shifting to another angle, it is important to analyze the safety profiles of biologic therapies for severe asthma, revealing both known and less-documented adverse events. While these therapies have transformed asthma management, the balance between efficacy and safety remains crucial. Limited long-term safety data underscore the need for enhanced post-market surveillance [17]. The link between omalizumab and eosinophilic granulomatosis with polyangiitis (EGPA) was also examined, suggesting its potential as a corticosteroid-sparing agent for severe asthmatic EGPA patients. However, conflicting evidence warns against hasty corticosteroid tapering, especially in refractory cases. There is a hint of a possible association between omalizumab use and increased EGPA risk, possibly linked to steroid reduction, though mechanisms remain uncertain [18]. It is important to note that there have been intriguing findings regarding the onset of eosinophilic granulomatosis with polyangiitis (EGPA) during treatment with anti-T2 monoclonal antibodies (mAbs) for severe asthma. It is also essential to contextualize these results within individual patient experiences. Notably, in our patient, some observed features, such as the increase in blood eosinophil level, the presence of obstructive airway disease and rhinitis symptoms, may have raised the suspicion of EGPA. We did not perform the testing for antineutrophilic antibodies; however, the maximum level of blood eosinophils was <1000 cell/μL, there were no clinical signs of nasal polyposis or vasculitis, and the chest X-ray was clear. Nevertheless, the importance of vigilance in monitoring patients receiving anti-T2 mAbs for potential development of EGPA must be underscored [19].

Collectively, these findings underscore the intricate and variable nature of T2-high asthma phenotypes, emphasizing the importance of targeted therapies tailored to specific inflammatory pathways for optimal asthma management.

## 4. Conclusions

In conclusion, attaining clinical remission is a viable outcome for individuals grappling with severe, uncontrolled asthma, especially in the age of biologic anti-asthmatic medications. To optimize the effectiveness of such therapies, it is imperative to conduct meticulous and careful patient phenotyping for every patient with severe asthma. This process, coupled with diligent monitoring throughout the therapeutic course, plays a pivotal role in either confirming positive progress or identifying instances of loss of control, as well as possible side effects of the biologic treatment. In cases, where necessary, a judicious switch to an alternative biologic agent may be warranted to ensure sustained and favorable outcomes in the management of severe asthma.

## Figures and Tables

**Table 1:** Favorable clinical evolution under anti-IgE biologic therapy.

	30 June 2020	24 July 2020	14 September 2020	15 October 2020	26 January 2021
**Score ACT**	22	24	24	23	24
**FEV1, L (%)**	1.40 (64%)	-	1.38 (63%)	1.35 (62%)	-
**Eosinophils (cells/μL)**	340	-	360	-	-
**Exacerbations**	0	0	0	0	0

**Table 2:** Deterioration of asthma control and onset of exacerbations alongside blood eosinophil increase.

	27 April 2021	23 June 2021	28 September 2021	17 November 2021	21 December 2021
**Score ACT**	19	19	13	24	19
**FEV1, L (%)**	1.15 (55%)	-	0.77 (37%)	1.54 (74%)	1.21 (58%)
**Eosinophils (cells/μL)**	500	-	710	-	520
**Exacerbations (CSO)**	1	1	1	0	0

**Table 3:** Monitoring evolution under benralizumab treatment.

	31 January 2022	2 March 2022	6 June 2022	2 September 2022	29 November 2022	10 February 2023	26 May 2023	18 October 2023	22 February 2024	24 April 2024
**Score ACT**	24	24	24	24	24	19	23	22	24	24
**FEV1, L (%)**	-	1.73 (80%)	1.51 (70%)	1.68 (78%)	1.71 (79%)	1.68 (78%)	1.68 (78%)	1.66 (77%)	1.51 (70%)	1.91 (88%)
**Eosinophils (cells/μL)**	-	-	0	-	330	290	500	510	300	-
**Exacerbations (CSO)**	0	0	0	0	0	1	0	0	0	0
**Adverse Effects**	0	0	0	0	0	0	0	0	0	0

## Data Availability

The original contributions presented in this study are included in the article. Further inquiries can be directed to the corresponding author.
